# Psychiatric Manifestations Associated with Aqueductal Stenosis-related Hydrocephalus: A Case Report

**DOI:** 10.1192/j.eurpsy.2026.10448

**Published:** 2026-07-31

**Authors:** I. Ilahi, A. Barkati, M. Othmani, H. Maktouf, A. Ben Hamadi, L. Mnif

**Affiliations:** Psychiatry F, Razi Hospital, Manouba, Tunisia

## Abstract

**Introduction:**

Hydrocephalus due to aqueductal stenosis is classically associated with neurological and cognitive symptoms. However, psychiatric manifestations, including psychotic episodes, remain underrecognized and poorly documented.

**Objectives:**

We report the case of a patient with aqueductal stenosis whose psychiatric presentations revealed and recurred during the course of the disease.

**Methods:**

We describe a 40-year-old male, with no family history of psychiatric or organic disorders, who initially presented in 2006 with a brief psychotic episode leading to the diagnosis of obstructive hydrocephalus by aqueductal stenosis. He underwent ventriculostomy with favorable outcome and significant improvement of psychiatric symptoms under olanzapine and valproate, discontinued after several months. The patient remained asymptomatic with good psychosocial functioning for almost two decades. In September 2025, he was admitted under involuntary hospitalization for acute psychomotor agitation and incoherent speech.

Psychiatric evaluation revealed a well-systematized delusional syndrome with grandiose and persecutory themes, interpretative and intuitive mechanisms. Neurological examination showed no focal deficit, except for a sluggish left pupillary light reflex. Brain MRI confirmed stable obstructive supratentorial hydrocephalus with patent ventriculostomy.

**Results:**

This case highlights the potential for recurrent psychotic manifestations in patients with long-standing hydrocephalus due to aqueductal stenosis, even in the absence of acute neurological deterioration. It underscores the need for systematic psychiatric monitoring in patients with obstructive hydrocephalus, particularly when initial presentation involves psychosis. The interplay between structural brain alterations and psychiatric vulnerability warrants further investigation.

**Conclusions:**

Hydrocephalus by aqueductal stenosis can manifest with isolated or recurrent psychotic symptoms, sometimes preceding or persisting despite neurosurgical treatment. Clinicians should maintain a high index of suspicion and adopt an integrated neuropsychiatric approach in the long-term follow-up of these patients.

**Disclosure of Interest:**

None Declared

